# Shields for Emotional Well-Being in Chinese Adolescents Who Switch Schools: The Role of Teacher Autonomy Support and Grit

**DOI:** 10.3389/fpsyg.2019.02384

**Published:** 2019-10-18

**Authors:** Xiaoyu Lan, Lifan Zhang

**Affiliations:** ^1^Faculty of Psychology, Beijing Normal University, Beijing, China; ^2^Department of Developmental Psychology and Socialization, University of Padua, Padua, Italy

**Keywords:** emotional well-being, teacher autonomy support, grit, school switching, Chinese adolescents

## Abstract

Although prior research has demonstrated that switching schools poses a risk for academic and behavioral functioning among adolescents, relatively little is known about their emotional adjustment, or how it affects emotional well-being. Moreover, the cumulative effects of multiple risk and protective factors on their emotional well-being are even less covered in the existing literature. Guided by a risk and resilience ecological framework, the current study compared emotional well-being, operationalized as positive affect and negative affect, between Chinese adolescents who had switched schools and their non-switch counterparts, and examined the direct and interactive effects of teacher autonomy support and two facets of grit (i.e., perseverance and consistency) on emotional well-being in both groups. A propensity score matching analysis was used to balance the two groups in terms of sociodemographic characteristics (i.e., age, gender, and socioeconomic status). A total of 371 adolescents who had switched schools and 742 non-switch counterparts aged from 13 to 18 years were involved in this study. Results indicated that adolescents who had switched schools reported higher levels of negative affect than their non-switch counterparts. Moreover, for adolescents who had switched schools, those who possessed higher levels of perseverance had a significantly negative association between teacher autonomy support and negative affect; however, the corresponding association was independent of perseverance for their non-switch counterparts. The current findings indicate that switching schools is a disadvantage for adolescents’ emotional states. However, teacher autonomy support and perseverance can protect adolescents who switch schools as critical stress-buffering factors against these negative feelings.

## Introduction

Educators and researchers have long voiced concern about the effects of switching schools on adolescents’ adjustment and achievement (for a review, see Welsh, 2017), and accordingly, the consequences of switching schools have been the focus of separate lines of research. Based on these findings, the majority of research has indicated that switching schools is associated with substantial academic and behavioral difficulties, such as low academic grades, high rates of dropping out, and behavior dysregulation (e.g., Mehana and Reynolds, 2004; Gasper et al., 2012), because switching schools requires adolescents to adjust to a new learning environment and to reconstruct peer networks and teacher-student relationships (Rumberger et al., 1999). Despite such research efforts in the last decades, gaps in knowledge remain. First and foremost, understanding of the outcomes beyond academic and behavioral variables, such as emotional well-being, appears relatively lacking, and this gap is particularly glaring during adolescence because this period of life is accompanied by psychological, physical, and social transformations, which causes adolescents to experience more frequent and intense emotions than children and adults (Larson and Lampman-Petraitis, 1989; Radloff, 1991). Second, some findings have argued that switching schools is not always harmful (Welsh, 2017). For example, switching to a higher quality school that provides better educational resources may offset and outweigh negative effects on adjustment in adolescence (Welsh, 2017). Although several attempts have been made to explain this paradox, studies on the cumulative effects of multiple risk and protective factors on their adjustment are still sparse (e.g., Bailey and Baines, 2012). Given that switching schools is an ecological transition encompassing some changes embedded in a person-environment interaction, the addition of literature exploring multiple risk and protective factors on emotional well-being is assumed to be potentially valuable for adolescents who had switched schools.

To fill these gaps, we used an integrative framework that combines an ecological perspective (Bronfenbrenner, 1979) with a risk and resilience perspective (Fergus and Zimmerman, 2005) to investigate possible risk and protective factors for adolescents’ emotional well-being. Following this approach, adolescents are embedded within layers of environmental systems, and development unfolds through the dynamic interactions between the individual and multiple contexts: the contextual level (e.g., teacher autonomy support) and the individual level (e.g., grit). As this framework demonstrates, the contextual or individual factor may serve as both an asset factor (showing a positive main effect) and a protective factor (by moderating the effect of a contextual or individual risk factor). Instead of examining asset or protective factors individually, it may be more informative to investigate how multiple factors (across domains or contexts) jointly or interactively shape adolescents’ developmental outcomes (Zhou et al., 2012). This approach has been successfully applied to identify the cumulative risk and protective factors for emotional adjustment (Lan et al., 2019c). Moreover, given the gaps in the existing literature, it is imperative and imminent that much attention may be paid to some contexts with high rates of switching schools, such as China.

With the rapid development of its economy in the last decades, China has witnessed a significantly increased rate of switching schools among adolescents. That is mainly due to the following situations. First, parents initiate switching of schools to let their school-aged children achieve a better educational fit and find a better school or community situation (Wu, 2013; Dong and Li, 2019); in this context, parents often mobilize their cultural, social, and economic capitals to guarantee that their children can attend key state schools in China (Wu, 2013). Second, unplanned moves are made in reaction to some situation in the family or school. For example, to achieve a better life standard and job opportunities, adolescents are forced to switch schools because of their parents’ residential or workplace mobility. Nevertheless, switching schools, in the context of Chinese culture, may be harmful to adolescents’ adjustment. For instance, social harmony and positive personal interactions are emphasized (Bond, 1996), and the disruption to social networks due to switching schools may pose a risk for adolescents’ emotional adjustment. Moreover, a successful adjustment at school in China is highly emphasized due to the longstanding fact that adjustment reflects on family dignity (Lan et al., 2019b). Over the past decades, although there has been a proliferation of policies in China to facilitate educational equity, little empirical attention has been paid to further understand adolescents’ emotional well-being after switching schools. Given the high rates of switching schools in China and potentially unfavorable outcomes adolescents may encounter, the current study seems relatively valuable in terms of providing insight into education policy and designing intervention or prevention programs that can mitigate the negative impacts of switching schools on adolescents’ emotional adjustment.

To briefly summarize, the current study aimed to compare emotional well-being of Chinese adolescents who had switched schools to their non-switch counterparts. Given the potential vulnerability of adolescents, we also aimed to explore the direct and interactive effects of teacher autonomy support and grit on facilitating emotional well-being, and these associations were expected to be more pronounced in adolescents who had switched schools. This is because a protective factor is more salient in the context of vulnerability (Fergus and Zimmerman, 2005; Lan et al., 2019c). The following sections provide a literature review to summarize the potential associations of teacher autonomy support and grit with emotional well-being.

### Teacher Autonomy Support and Emotional Well-Being

In the current study, we focused on emotional well-being, indexed by positive affect and negative affect. This was done to echo the paradox of switching schools in adolescence, because prior research has suggested both positive and negative outcomes as a result of switching schools. Moreover, previous studies indicate that positive affect and negative affect are independent components in terms of how much individuals feel in their lives over longer time periods (Diener and Emmons, 1984; Larsen et al., 2017), and thus both positive and negative outcomes allowed us to comprehensively capture adolescents’ emotional adjustment.

Adolescence is a time of many developmental and life changes, such as increases in autonomy-seeking. According to self-determination theory (SDT; Ryan and Deci, 2000), individuals naturally tend to self-organize their own actions, and the sense of choice that characterizes autonomy is a necessary aspect of well-being. As such, autonomy support from significant others appears critical to improving adolescents’ emotional well-being, which is one of the main focuses of the present research. Prior research about the correlates of emotional well-being in Chinese adolescents has shown that perceived social support (e.g., teacher support and classmate support) is positively linked to adolescents’ subjective well-being, and teacher support shows a stronger association with well-being than classmate support (Liu et al., 2016; Tian et al., 2016). Indeed, considering the large amount of time that adolescents spend at school and the critical role of teachers during this period, it seems logical that teacher autonomy support would be more influential in facilitating adolescents’ emotional well-being than support from classmates. Therefore, from a social-environmental perspective, the current study centered on the role of teacher autonomy support on emotional well-being.

Teacher autonomy support refers to the teachers in the classrooms providing a meaningful rationale, acknowledging negative feelings, using non-controlling language, offering meaningful choices, and nurturing internal motivational resources for their students (Núñez and León, 2015). Prior research has documented that when teachers become more supportive of autonomy, their students show wide-ranging gains in adaptive functioning, including well-being (Vansteenkiste et al., 2012; Cheon et al., 2018). This is mainly because the autonomy-supportive motivating style in the classroom catalyzes engagement-fostering motivations, which in turn facilitate students’ adaptive functioning. In the context of Chinese culture, a burgeoning body of research has also highlighted the positive role of teacher autonomy support on Chinese adolescents’ optimal functioning (e.g., Yu et al., 2016; Wang et al., 2017). Particularly, Yu et al. (2016) have uncovered that teacher autonomy support can significantly reduce anxiety and depression in Chinese adolescents, suggesting that teacher autonomy support may be critical in facilitating well-being in Chinese adolescents. On the basis of theoretical perspective and empirical findings, we assume that teacher autonomy support is positively associated with positive affect, and negatively associated with negative affect in Chinese adolescents.

### Grit

From an individual characteristic perspective, the variable selection was informed by self-regulatory theory (Carver and Scheier, 2004; de Ridder and de Wit, 2006). Within this theory, self-regulatory traits refer to efforts by humans to alter their thoughts, feelings, desires, and actions in the pursuit of goals. Central to Carver and Scheier’s approach, goals orientation has the potential to induce positive affect and negative affect. According to this approach, we propose that grit as a self-regulatory trait may fulfill our research purpose.

Grit involves perseverance and passion for long-term goals in the face of challenging circumstances (Duckworth et al., 2007). To date, most of the literature related to grit has found that grit is negatively associated with negative emotional states, such as depression (e.g., Datu et al., 2018a), and positively associated with academic performance (e.g., Datu et al., 2018b). In separate lines of research, grit is found to be moderately associated with other self-regulatory traits, such as conscientiousness and self-control (e.g., Ivcevic and Brackett, 2014). However, we assume that grit is more appropriate to fulfill our research objectives than other self-regulatory traits, due to the following empirical and cultural considerations. First, aligned with self-regulation theory (de Ridder and de Wit, 2006), adaptive competencies draw on longer volitional processes of goal striving, whereas conscientiousness and self-control refer to short-term goal orientations (Duckworth and Gross, 2014). Second, given the potential vulnerability of the students who switch schools, an emerging body of research highlights the protective role of grit in adolescents’ socioemotional adjustment, especially under an unfavorable condition. For example, Lan and Moscardino (2019) found that in the context of negative teacher-student relationships, high levels of grit can buffer student well-being in Chinese adolescents. Third, Chinese society emphasizes the protective roles of diligence and perseverance when individuals encounter adversities and challenges (Lan et al., 2019a). On the basis of the literature reviewed above, we assume that grit is positively associated with positive affect, and negatively associated with negative affect.

Furthermore, grit consists of two facets (Duckworth et al., 2007): perseverance of effort (hereafter, “perseverance”) and consistency of interests (hereafter, “consistency”). The former describes the extent to which individuals can endure setbacks and difficulties while sustaining personal effort, whereas the latter refers to the degree to which individuals continuously concentrate on achieving their long-term aspirations (Duckworth et al., 2007; Datu et al., 2016b). However, a recent meta-analysis has questioned the construct validity of grit (Credé et al., 2017), demonstrating that the utility of grit mainly depends on perseverance but not consistency. Meanwhile, Datu et al. (2016a) found that perseverance fits into a collective cultural context well, but consistency does not. Additionally, research examining the roles of perseverance and consistency suggests that these two facets play different roles in emotional states. For example, Datu and Fong (2018) found that Chinese primary school students with high perseverance and low consistency show high positive activating emotions (e.g., hope) and reduced levels of negative activating emotional states (e.g., anxiety and shame). Likewise, Disabato et al. (2019) demonstrated that perseverance is moderately associated with subjective well-being and personality strengths, whereas consistency is weakly and negatively related to these outcomes. Given the differential roles of perseverance and consistency, we distinguish two facets of grit when examining the association of grit with emotional well-being.

### The Present Study

To sum up, the current study had two main goals: (a) to compare emotional well-being in adolescents who switch schools and their non-switch counterparts, and (b) to examine the direct and interactive effects of teacher autonomy support and the two facets of grit on emotional well-being in both groups. Moreover, previous research has shown that sociodemographic characteristics are potentially related to our dependent variables in Chinese adolescents. For example, females show higher levels of well-being than males (Chen et al., 2016); older aged adolescents report lower levels of well-being than younger adolescents (Liu et al., 2016); and SES (socioeconomic status) is positively associated with well-being (Ni et al., 2016). Taken together, this study regarded age, gender, and SES as potential covariates. Specifically, we tested the following hypotheses (H):

(H1) After controlling for sociodemographic characteristics, adolescents who switch schools report higher levels of negative affect and lower levels of positive affect than their non-switch counterparts.

(H2) After controlling for sociodemographic characteristics, teacher autonomy support and the two facets of grit are positively associated with positive affect and negatively related to negative affect.

(H3) After controlling for sociodemographic characteristics, adolescents reporting higher levels of teacher autonomy support, perseverance, and/or consistency score higher on positive affect and lower on negative affect than adolescents reporting lower levels of perseverance and/or consistency (i.e., two-way interaction; H3a), and these associations are stronger for adolescents who switch schools (i.e., three-way interaction; H3b).

**FIGURE 1:**
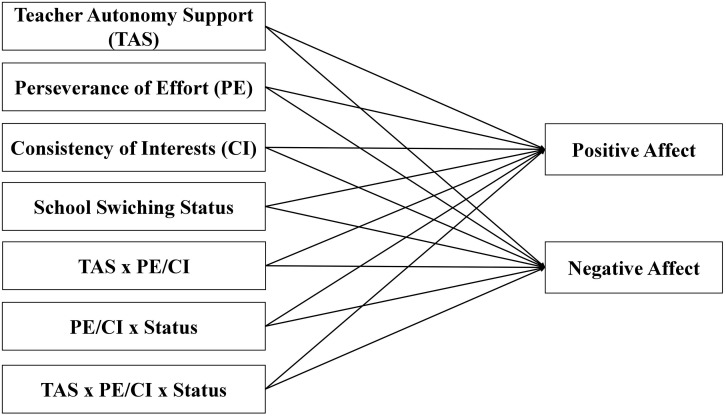
A hypothesized model. Age, gender, and socioeconomic status were considered as covariates.

A graphical representation of our hypothesized model is depicted in Figure 1.

## Materials and Methods

### Participants

The current study was based on a project entitled “Socioecological Correlates of Psychosocial and Academic Functioning in Chinese Adolescents.” We recruited participants from four grades (7th, 8th, 10th, and 11th) within 10 public middle and high schools located in different regions of north mainland China (i.e., Harbin, Lanzhou, and Beijing) through personal networks. We did not include 9th and 12th graders, because they may encounter high pressure for entrance examination during the last year of middle and high schools. Finally, approximately 2,700 students in those schools agreed to participate, and 13.7% (*n* = 371) adolescents who had switched schools were identified through an archival review of school records. To obtain a comparable sample with similar characteristics (i.e., gender, age, and SES), we conducted a propensity score matching analysis, as suggested by pertinent research about adolescents who switch schools (Gasper et al., 2012). Although Gasper et al. suggested a ratio of one-to-one is assumed to be sufficient, we adopted a ratio of one-to-two in order to ensure enough statistical power, given the sample size of adolescents who had switched schools in the present research. The same ratio can be found in prior research (e.g., Lan et al., 2019a). Participants in this study comprised 371 adolescents who switched schools (60.1% girls) and 742 or their non-switch (60.1% girls) counterparts, aged 13–18 years (*M*_*age*_ = 15.87; *SD* = 1.52). As for adolescents who had switched schools, most of their fathers (54.7%) had completed high school education, and the majority of their mothers (30.5%) had finished middle school education. For their non-switch counterparts, most of their fathers (56.3%) and most of their mothers (56.1%) had completed high school education.

### Procedure

Prior to data collection, ethical approval for this study was granted by the principal investigator of the university and collaborative schools. Through personal networks, the authors and research assistants contacted the public primary and secondary schools located in different regions of north mainland China. Those regions were the northeast (i.e., Harbin, Heilongjiang Province), northwest (i.e., Lanzhou, Gansu Province), and north (i.e., Beijing) of mainland China. These schools are all located in urban areas, and only adolescents with urban household registrations are entitled to attend. After obtaining permission from school principals, a project brochure and an informed consent form were sent to the head teacher in each classroom. Suggested by prior research (Wang et al., 2019), the head teachers were asked to send a message to inform parents about the purpose and voluntary nature of the survey in the Parents WeChat Group. Only on the condition that all parents approved would the children be allowed to participate in the current project. In the meantime, a verbal agreement was obtained from each adolescent. In total, participation rate was approximately 95%, which is in accordance with prior research of Chinese adolescents (e.g., Lan et al., 2019a). Eligibility criteria in the present research were as follows: (a) the adolescents were between 13 and 18 years old; (b) those who had transferred to another school at least once during primary and secondary school, either within one city or between two cities in mainland China (for adolescents who had switched schools only); and (c) the type of school switching was limited to non-structural mobility, defined as unplanned moves made in reaction to some situation in the family or school, or purposeful and planned moves made to achieve a better educational fit and to find a better school or community situation (for adolescents who had switched schools only; Welsh, 2017). This was done to differentiate from structural mobility, which refers to the scheduled transition from primary to middle school or from middle to high school that is dependent on the structural change inherent in the education system. During school hours, a trained research assistant provided standardized instructions (i.e., confidentiality and anonymity of participation, right to withdraw and debrief), and participants were asked to complete the questionnaires during a regular class hour.

### Measures

Sociodemographic characteristics were provided by several self-report questions, such as age, gender, parental education level and occupation, and monthly family income. SES was measured by parental education level and occupation, and family income per month. In terms of parental education, four options were provided: (a) middle school graduation or less, (b) high school graduation, (c) bachelor’s degree graduation, and (d) master’s degree graduation or higher. Moreover, seven choices were available for parental occupation and monthly family income based on Chinese occupational classifications and income criteria. Overall, the three scores were standardized and summarized to yield an SES score, with higher values indicating higher SES (Lan and Moscardino, 2019).

The histories of switching schools were collected through an archival review of school records. To double-check the accuracy of this information, adolescents were also asked to report their experiences of switching schools in terms of the overall number of schools they attended during their primary and secondary school years.

Teacher autonomy support was assessed using a subscale of the Learning Climate Questionnaire (LCQ; Black and Deci, 2000). This subscale consists of nine items. One of the examples is “I feel that my teacher provides me choices and options.” Participants were asked to assess each item on a 7-point Likert scale ranging from 1 (*strongly disagree*) to 7 (*strongly agree*). The mean score was yielded to represent the score of teacher autonomy support, with higher values indicating higher levels of perception of autonomy support from teachers. Previous research has demonstrated good internal consistency for this scale in Chinese adolescents (e.g., Vansteenkiste et al., 2012). In the present study, this scale showed good reliability and validity [Cronbach’s alpha was 0.92 for both groups; χ^2^(27) = 222, *p* < 0.001; the non-normed fit index (NNFI) = 0.95; the comparative fit index (CFI) = 0.96; the root mean square error of approximation (RMSEA) = 0.08].

Grit was measured by the 8-item Grit Scale (Duckworth and Quinn, 2009). This scale was validated in Chinese adolescents by Li et al. (2018b), showing good validity and reliability. This scale contains two dimensions: perseverance (four items; e.g., “Setbacks do not discourage me”) and consistency (four items; e.g., “New ideas and projects sometimes distract me from previous ones”). Participants were asked to rate each item from 1 (*not like me at all*) to 5 (*very much like me*) on a Likert scale. The average score of the corresponding items was calculated separately to yield the score of perseverance and consistency, with a higher value indicating higher levels of perseverance and consistency. In the current study, Cronbach’s alphas for perseverance were 0.81 and 0.79 for adolescents who switch schools and their counterparts, respectively. In terms of consistency, Cronbach’s alpha was 0.80 for both groups. Moreover, as prior research has raised issues about the psychometric validity of the Grit Scale, especially in non-Western contexts (e.g., Datu et al., 2016a), confirmatory factor analysis was used to ensure the construct validity of grit in the current study. Results showed an acceptable model fit: χ^2^(19) = 161, *p* < 0.001; NNFI = 0.93; CFI = 0.95; RMSEA = 0.08.

Positive and negative affect were measured by the 14-item Affect Balance Scale (ABS; Bradburn, 1969). ABS has been used to assess Chinese adolescents by Yang et al. (2017), showing adequate properties. This scale consists of two dimensions: positive affect (eight items; e.g., “I feel particularly excited or interested in something”) and negative affect (six items; e.g., “I feel so restless that I could not sit long in a chair”). Participants were asked to rate each item from 1 (*never*) to 4 (*always*), based on their frequency of experiencing the given feeling. The average scores for positive affect and negative affect were calculated separately, with higher values indicating higher levels of positive affect and negative affect. A previous study has reported good internal consistency of this scale (Yang et al., 2017). In the present study, Cronbach’s alphas were 0.84 and 0.83 for positive affect in adolescents who switch schools and their counterparts, respectively. For negative affect, coefficients were 0.77 and 0.79, respectively. Moreover, results of confirmatory factor analysis showed an acceptable model fit of the ABS in the current study: χ^2^(76) = 549, *p* < 0.001; NNFI = 0.89; CFI = 0.90; RMSEA = 0.07.

### Data Analyses

Data analyses were performed using SPSS 21.0 (IBM Corp, 2012) and R software (R Core Team, 2017). Twenty cases were excluded because we did not obtain the information regarding their experiences of switching schools. In addition, eight cases were omitted due to high rates of missing data (more than 20%). This procedure was done before we conducted a propensity score matching analysis. To investigate the impact of missing data (less than 20%), we performed a Little’s Missing Completely at Random (MCAR) test. Results supported the MCAR assumption, χ^2^(105) = 101.83, *p* = 0.57. Therefore, full-information maximum likelihood estimates were employed to impute missing data.

Moreover, suggested by prior research (Wang et al., 2019), we conducted Harman’s single-factor test to evaluate the potential common method bias in the current study. This was done because this study heavily relied on self-reported measurement, which may potentially be affected by response bias. As such, all items in this study were loaded into an exploratory factor analysis and the results indicated the presence of five factors with initial eigenvalues greater than 1.00. The first factor accounted for 23.43% of the variance, suggesting that the influence of common method variance was relatively small (Podsakoff et al., 2003).

Regarding our research purposes, descriptive information for the sample was summarized using means and standard deviations for continuous variables. Pearson’s correlations were used to evaluate associations among the study variables. To examine group differences in the two outcome variables, Multivariate Analysis of Covariance (MANCOVA) was used. Moreover, we used path analyses for observed variables to evaluate the direct and interactive contributions of teacher autonomy support and two facets of grit to positive affect and negative affect in Chinese adolescents. Our hypothesized model was tested using the R package lavaan (Rosseel, 2012; R Core Team, 2017). To evaluate the goodness of fit of the model, several indices were taken into consideration: χ^2^, NNFI, CFI, and RMSEA (Schermelleh-Engel et al., 2003). For these indices, values of NNFI and CFI higher than 0.95 and 0.97, respectively, and values of RMSEA lower than 0.05 can be considered a good fit. Path coefficients from teacher autonomy support to positive affect and negative affect were estimated using the maximum likelihood method, with a single observed score (i.e., centered mean score) for each variable. To test for moderation, products between centered variables were computed and included in the model as interaction terms (Lan and Radin, 2019).

## Results

### Descriptive Statistics

Means and standard deviations for study variables and bivariate correlations are reported in Table 1, separately for adolescents who had switched schools and their non-switch counterparts.

**TABLE 1:** Descriptive statistics and bivariate correlations of study variables for adolescents who had switched schools and their non-switch counterparts.

	**Switch (*n* = 371)**			**Non-switch (*n* = 742)**										
	***M***	***SD***	**Range**	***M***	***SD***	**Range**	**1**	**2**	**3**	**4**	**5**	**6**	**7**	**8**
1. TAS	3.59	0.83	1–5	3.74	0.77	1–5	–	0.27^∗∗∗^	0.09^∗^	0.25^∗∗∗^	–0.17^∗∗∗^	0.06	0.001	–0.001
2. PE	3.53	0.85	1–5	3.64	0.82	1–5	0.30^∗∗∗^	–	0.39^∗∗∗^	0.32^∗∗∗^	–0.27^∗∗^	–0.11^∗∗^	–0.13^∗∗∗^	0.06
3. CI	2.98	0.93	1–5	3.03	0.88	1–5	0.15^∗∗^	0.37^∗∗∗^	–	0.14^∗∗∗^	–0.27^∗∗∗^	–0.06	0.05	–0.01
4. PA	3.20	0.51	1–4	3.23	0.49	1–4	0.31^∗∗∗^	0.30^∗∗∗^	0.14^∗∗^	–	–0.32^∗∗∗^	0.01	0.12^∗∗∗^	0.01
5. NA	2.46	0.59	1–4	2.37	0.60	1–4	–0.10	–0.22^∗∗∗^	–0.27^∗∗∗^	–0.29^∗∗∗^	–	0.10^∗∗^	0.08^∗^	−0.08^∗^
6. Age	15.86	1.51	13–18	15.88	1.52	13–18	–0.08	–0.14^∗∗^	–0.03	−0.11^∗^	0.05	–	–0.01	0.10^∗∗^
7. Gender^a^	–	–	1–2	–	–	1–2	0.001	–0.09	–0.01	0.12^∗^	0.01	0.02	–	–0.05
8. SES	0.12	3.61	−8.12–10.70	–0.10	3.63	−9.09–11.48	–0.06	0.13^∗^	–0.02	–0.04	0.03	0.05	–0.07	–

*Correlation coefficients displayed above the diagonal are for adolescents who had not switched schools, below for adolescents who had switched schools. TAS = teacher autonomy support, PE = perseverance of effort, CI = consistency of interests, PA = positive affect, NA = negative affect, and SES = socioeconomic status. ^*a*^Coded as 1 = male, 2 = female. ^∗^*p* < 0.05, ^∗∗^*p* < 0.01, and ^∗∗∗^*p* < 0.001.*

As shown in Table 1, the results indicated that teacher autonomy support and two facets of grit were each significantly and positively associated with positive affect, and negatively related to negative affect in both groups.

Multivariate analysis of covariance—after controlling for age, gender, and SES—indicated that adolescents who had switched schools reported higher levels of negative affect, *F*(1, 1108) = 6.45, *p* = 0.01, partial η^2^ = 0.01, in comparison to their non-switch counterparts, but there was no significant difference in positive affect, *F*(1, 1108) = 0.85, *p* = 0.36, between adolescents who switched schools and their non-switch counterparts.

### Path Analyses

**FIGURE 2:**
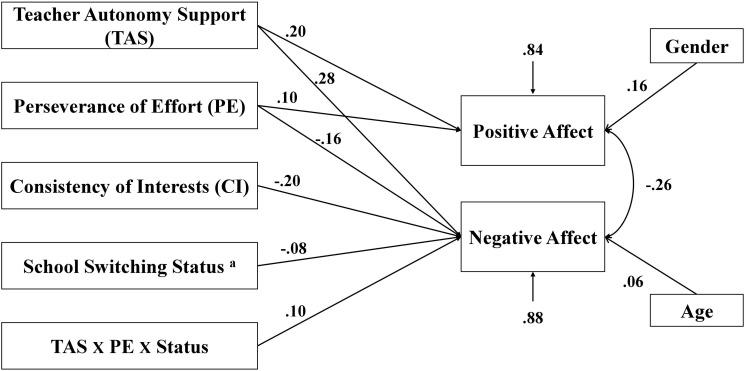
Standardized path coefficients for the final path model. ^a^Coded as 1 = adolescents who had switched schools, 0 = adolescents who had not switched schools. All coefficients were significant with *p* < 0.05.

First, the baseline model was tested (see Figure 1), and inspection of path coefficients showed many non-significant links between interaction terms and outcome variables. For the sake of parsimony, these links were removed step by step based on *p*-value, and the model was re-evaluated. The final model, presented in Figure 2, fit the data well [χ^2^(5) = 3.12, *p* = 0.68; NNFI = 0.99; CFI = 0.99; RMSEA < 0.01]. The *R*^2^ for the endogenous variables indicated that the model accounted for 16.2% of the variance in positive affect and 12.1% of the variance in negative affect.

As shown in Figure 2, teacher autonomy support and perseverance were each significantly and positively associated with positive affect. Moreover, teacher autonomy support, perseverance, consistency, and switching school status were significantly and negatively related to negative affect. Furthermore, the interaction term among teacher autonomy support, perseverance, and switching school status was positively associated with negative affect.

**FIGURE 3:**
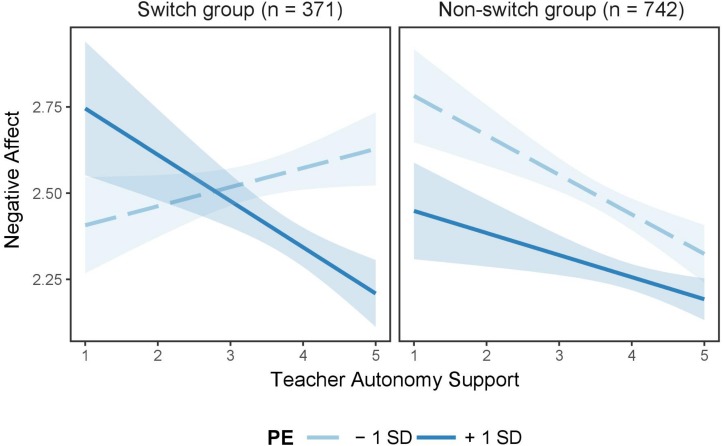
Interaction effect of teacher autonomy support and perseverance of effort on negative affect for adolescents who had switched schools (*n* = 371) and their non-switch counterparts (*n* = 742). Perseverance of effort (PE) was divided into two levels based on mean: low = *M* – 1*SD*, high = *M* + 1*SD*. Corresponding bands refer to 95% confidence intervals.

Moreover, simple slope analysis showed that for adolescents who had switched schools, the association between teacher autonomy support and negative affect was significant at high levels of perseverance (*B* = −0.13, *SE* = 0.05, *t* = −2.59, *p* < 0.01) but not at low levels of perseverance (*B* = 0.06, *SE* = 0.04, *t* = 1.28, *p* = 0.20). However, the association between teacher autonomy support and negative affect was significant at both low levels of perseverance (*B* = −0.11, *SE* = 0.04, *t* = −2.91, *p* < 0.001) and high levels of perseverance (*B* = −0.06, *SE* = 0.04, *t* = −1.81, *p* = 0.05), indicating that this association was independent of perseverance in adolescents who had not switched schools (see Figure 3).

## Discussion

The goals of this study were to compare emotional well-being operationalized by positive affect and negative affect in Chinese adolescents who had switched schools and their non-switch counterparts, and to examine the associations of teacher autonomy support and two facets of grit with emotional well-being. Moreover, guided by a risk and resilience ecological framework, the potential two- and three-way interaction effects among teacher autonomy support, grit, and school switching status on emotional well-being were investigated. Although extant research suggests that switching schools is a potential risk factor for adolescent academic and behavioral adjustment, relatively little is known about emotional adjustment, such as emotional well-being; moreover, the cumulative effects of multiple risk and protective factors on emotional well-being is less explored in the existing literature. Our findings showed that adolescents who had switched schools reported higher levels of negative affect than their non-switch counterparts, but there were no significant differences in positive affect between the two groups. Moreover, teacher autonomy support and perseverance were positively correlated with positive affect, and negatively associated with negative affect; consistency was negatively associated with negative affect. Additionally, in the context of higher levels of teacher autonomy support, higher levels of perseverance can mitigate negative affect for adolescents who switch schools.

The first goal of this study was to compare positive affect and negative affect in both groups. In line with the first hypothesis, adolescents who had switched schools reported higher levels of negative affect than their counterparts. This finding is in accordance with previous research, confirming that adolescents who switch schools experience higher levels of emotional disturbance (Malmgren and Gagnon, 2005). Non-structural school switching is complex and driven by a confluence of social and economic factors, resulting in a discontinuity in early learning environments. This is against the assertion highlighting the importance of regularity and stability in early learning environments (Bronfenbrenner, 1979). Indeed, non-structural school switching often puts adolescents in a “minority” status in the classroom. They have to adjust to a new environment as well as reconstruct social relationships and peer group interactions. Moreover, Chinese culture attaches special importance to interdependence and social networks (Bond, 1996); as such, difficulties caused by the adjustment to a new context may bring a heightened negative effect on adolescents’ emotional well-being. However, contrary to the first hypothesis, there were no significant differences in terms of positive affect between the two groups. One possible explanation is the homogeneity of the two groups in this study. For instance, as documented by prior research (Ni et al., 2016), SES is positively related to positive affect. As such, the similar SES background across the two groups may minimize the difference in positive affect. Another possible interpretation is ascribed to the traditional Chinese cultural emphasis on emotional control and moderation (Soto et al., 2005), particularly in terms of positive feelings. These factors may help explain why adolescents from the two groups did not show a significant difference in positive affect.

The second aim of this study was to explore the associations of teacher autonomy support and two facets of grit with emotional well-being in Chinese adolescents. Aligned with the second hypothesis, the findings showed that teacher autonomy support was positively correlated with positive affect, and negatively associated with negative affect. One possible explanation is attributable to the role of teachers in Chinese society. Given the majority of time school-aged adolescents spend at school, teachers are often regarded as the main influential or guiding role in youth emotional adjustment (Ho et al., 2001; Li et al., 2018a). As such, teachers who offer an autonomy-supportive motivating style in the classroom can elevate engagement-fostering motivations, which in turn facilitate positive feelings and minimize negative feelings of adolescents (e.g., Cheon et al., 2018). Another possible explanation is informed by SDT (Ryan and Deci, 2000), which suggests that individuals are prone to self-organizing and self-selection, and that the satisfaction of autonomy need can facilitate positive adaptive functioning, such as emotional well-being. Moreover, our findings showed that perseverance was positively correlated with positive affect, and negatively associated with negative affect; consistency was negatively associated with negative affect. Such a finding corroborates previous research on the positive correlation between grit and positive affective states (e.g., Datu et al., 2016b, 2018b). However, consistency did not show a significant association with positive affect. This finding may be explained by prior research illustrating that perseverance is more salient in predicting subjective well-being than consistency in a collective setting (Datu et al., 2016a).

The third goal of this study was to ascertain the interactive associations of teacher autonomy support and two facets of grit with emotional well-being in both groups. Our findings showed that perseverance moderated the association between teacher autonomy support and negative affect in adolescents who had switched schools. To be specific, in adolescents who endorsed higher levels of perseverance, the association between teacher autonomy support and negative affect turned out to be significantly negative. One possible explanation is ascribed to the Chinese cultural highlight of perseverance, and perhaps individual motivation to persevere as a way to contribute to their families and school communities, which is independent of their personal interests (Disabato et al., 2019). Therefore, along with sufficient teacher autonomy support, individuals with high perseverance can mobilize various types of strength to buffer negative emotional states triggered by the experience of switching schools. However, this study did not show any significant interactive effects on positive affect. One possible explanation is that grit is more salient in negative conditions (Duckworth et al., 2007). For example, Lan and Moscardino (2019) have found that grit can buffer student well-being in the context of negative teacher-student relationships. As such, grit may not have a strong protective effect on positive outcomes. From this perspective, other self-regulatory traits (e.g., emotional regulation; Extremera and Rey, 2015) may help to explain positive outcomes. In addition, consistency did not show any interactive effects among study variables. One possible interpretation is that youth from a collective culture may show lesser tendencies to espouse consistent thoughts, emotions, and actions across different situations; instead, they are more likely to adopt a context-sensitive self (Datu et al., 2018b). Another explanation is aligned with the experience of switching schools, which requires better social integration skills in a new learning environment through making continuous efforts instead of continuously concentrating on achieving their long-term aspirations. Thus, the role of consistency is not salient in the association of teacher autonomy support with emotional well-being.

Overall, our findings went beyond traditional academic and behavioral outcomes by examining both positive and negative emotional outcomes in adolescents who switch schools, as compared with their non-switch counterparts. Based on the current findings, this study may refute the assertion that non-structural school switching may have some “benefits.” Moreover, the adaptability of the positive role of autonomy support among adolescents with various functioning levels in a collective setting remains under debate, as conformity and interdependence are highly embedded in collective societies (Markus and Kitayama, 1991). Along with other pertinent research (Yu et al., 2016), the current study further confirms the universally positive effect of autonomy support on adaptive functioning in adolescence in a collective context. Given the high rates of switching schools in China and the emotional vulnerability of adolescent who switch schools, the cumulative effects of teacher autonomy support and perseverance are highlighted as factors that mitigate negative affect. The current study may be particularly significant in the efforts to develop preventive aspects in the social environment (i.e., teacher autonomy support) and individual characteristics (i.e., grit) to facilitate adolescents’ emotional well-being during this transition.

### Limitations and Implications

Along with these significant findings, several limitations should also be acknowledged when interpreting the current findings. First, the current study relies on a cross-sectional design, which has less power than a longitudinal design when it comes to excluding time-invariants and unobserved individual differences, as well as in terms of observing a certain event’s temporal order. For example, this study fails to consider the impact of pre-transfer risk and resilience factors on well-being, despite, as documented by prior research, these factors being salient concerning well-being (e.g., Gasper et al., 2012). Given the growing incidence of school switching, further research adopting a longitudinal design to address the impact of school transfer over time may yield useful insights (Welsh, 2017). Second, the current study does not differentiate the timing, frequency, types, and distance involved in switching schools; however, previous studies indicate that these variables can covariate the deleterious effects of switching schools on adolescents’ adjustment (Pears et al., 2015; Welsh, 2017). Therefore, future study should unpack those effects to gain more credible estimates of the impact of switching schools. Third, although the effect of common method bias is proved to be small in the current study, self-report measurement may still fail to exclude the biases caused by response style and social desirability (Podsakoff et al., 2003). Moreover, teacher autonomy support is assessed by the perception of autonomy support, but not the actual autonomy being nurtured by teachers in the classroom. As such, future research should use a mixed-methods and multi-informant approach. Fourth, the current study reckons on a “narrow” operationalization of well-being, which highlights the affective aspects only. Future research initiatives should incorporate cognitive (i.e., appraised life satisfaction; Diener, 2000) and even eudaimonic aspects of well-being (Ryff and Singer, 2008), when examining the buffering roles of teacher autonomy support and perseverance. Fifth, although the current sample size may be sufficient, at least in terms of addressing our research questions and fulfilling the analytical approach, the small effect size revealed may imply that a larger sample size is optimal; moreover, given the size of the Chinese population and regional differences (north vs. south; rural vs. urban), a nationally representative sample should be used in any future study. Finally, the present research is built on a monocultural dataset, which precludes the possibility to generalize the current findings into other cultural contexts. As such, further investigation into the direct and interactive effects of teacher autonomy support and perseverance on well-being in both collective- and individual-focused contexts is essential.

Despite such limitations, the current study may have several theoretical and practical implications. With regard to theory, the current study confirms a risk and resilience ecological framework in the context of Chinese culture (Bronfenbrenner, 1979; Fergus and Zimmerman, 2005): switching schools is a risk factor for Chinese adolescents’ negative emotional states, and the ecological interactions between environmental and individual factors can mitigate this detrimental effect of school switching on adolescents’ emotional adjustment. Moreover, this study enriches SDT (Ryan and Deci, 2000): satisfaction of autonomy support can nurture and build youths’ inner motivation, which in turn can facilitate their emotional well-being. Additionally, this study corroborates self-regulation theory (Carver and Scheier, 2004; de Ridder and de Wit, 2006), suggesting that longer volitional processes of goal striving can facilitate adaptive competencies. From an applied perspective, this study suggests that the traditional methods of Chinese teachers (i.e., authoritative teachers) should be adjusted. In the classroom context, teachers should provide an atmosphere in which youths are not pressured to behave in a specific way; instead, teachers can motivate students by providing a meaningful rationale, acknowledging negative feelings, using non-controlling language, and offering meaningful choices (Núñez and León, 2015). As for adolescents who switch schools, some activities facilitating perseverance are beneficial in mitigating the detrimental effects of switching schools on their negative emotional states. For example, school educators and teachers should organize some activities with long-term goals that emphasize sustained effort despite the presence of setbacks and distress (Disabato et al., 2019; Lan and Moscardino, 2019).

## Data Availability Statement

The datasets generated for this study are available on request to the corresponding author.

## Ethics Statement

The studies involving human participants were reviewed and approved by the Ethics Committee at collaborative schools. Online informed consent to participate in this study was provided by the participants’ legal guardian/next of kin.

## Author Contributions

XL conceived this study, performed the statistical analyses, and drafted the manuscript. LZ assisted with the preparation of the manuscript. Both authors read and approved the final draft of the manuscript.

## Conflict of Interest

The authors declare that the research was conducted in the absence of any commercial or financial relationships that could be construed as a potential conflict of interest.
